# Equation-of-Motion Coupled-Cluster Cumulant Green’s Function for Excited States and X-Ray Spectra

**DOI:** 10.3389/fchem.2021.734945

**Published:** 2021-09-22

**Authors:** F. D. Vila, J. J. Kas, J. J. Rehr, K. Kowalski, B. Peng

**Affiliations:** ^1^ Department of Physics, University of Washington, Seattle, WA, United States; ^2^ Physical and Computational Science Directorate, Pacific Northwest National Laboratory, Richland, WA, United States

**Keywords:** coupled cluster, cumulant, green’s function, excited states, x-ray

## Abstract

Green’s function methods provide a robust, general framework within many-body theory for treating electron correlation in both excited states and x-ray spectra. Conventional methods using the Dyson equation or the cumulant expansion are typically based on the *GW* self-energy approximation. In order to extend this approximation in molecular systems, a non-perturbative real-time coupled-cluster cumulant Green’s function approach has been introduced, where the cumulant is obtained as the solution to a system of coupled first order, non-linear differential equations. This approach naturally includes non-linear corrections to conventional cumulant Green’s function techniques where the cumulant is linear in the *GW* self-energy. The method yields the spectral function for the core Green’s function, which is directly related to the x-ray photoemission spectra (XPS) of molecular systems. The approach also yields very good results for binding energies and satellite excitations. The x-ray absorption spectrum (XAS) is then calculated using a convolution of the core spectral function and an effective, one-body XAS. Here this approach is extended to include the full coupled-cluster-singles (CCS) core Green’s function by including the complete form of the non-linear contributions to the cumulant as well as all single, double, and triple cluster excitations in the CC amplitude equations. This approach naturally builds in orthogonality and shake-up effects analogous to those in the Mahan-Noizeres-de Dominicis edge singularity corrections that enhance the XAS near the edge. The method is illustrated for the XPS and XAS of NH_3_.

## 1 Introduction

The core-level x-ray absorption spectra (XAS) *μ*(*ω*) is typically described formally by Fermi’s golden rule. However, this formulation requires calculations of and summations over eigenstates of the many-body Hamiltonian *H* and is computationally intractable. Simplifications such as the determinantal ΔSCF approach, in terms of Slater determinants (Liang et al., 2017; Liang and Prendergast, 2019; Nozieres and Combescot, 1971) have similar limitations. Although still computationally demanding, Green’s function methods provide an attractive alternative since summation over final states is implicit (Lee et al., 2012; Bertsch and Lee, 2014; Rehr et al., 2009). Real-time approaches can also be advantageous as they avoid explicit calculations of eigenstates. Our treatment here exploits real-time approaches, following several recent developments: 1) Equation of motion coupled-cluster (EOM-CC) approaches for molecular systems have been formulated for the Green’s function in energy-space (Peng and Kowalski, 2016; Peng and Kowalski, 2018a), 2) An approach has also been developed (Rehr et al., 2020) for calculations of many-body XAS *μ*(*ω*) in terms of the convolution of a one-body XAS *μ*_1_(*ω*) and the spectral function of the core-hole *A*_*c*_(*ω*)

$$\mu \left(\omega \right)=\int dte^{-i\omega t}\mu \left(t\right)=\int d\omega^{\prime }\mu_{1}\left(\omega^{\prime }\right)A_{c}\left(\omega -\omega^{\prime }\right).$$ (1)

This result originates from the time-correlation approach (Nozieres and de Dominicis, 1969) that was used to solve the x-ray edge-singularity problem. In this approach the time-domain XAS transition amplitude *μ*(*t*) is given by the factorization

$$\mu \left(t\right)=\left\langle \Psi |D\left(0\right)D\left(t\right)|\Psi \right\rangle =L\left(t\right)G_{c}\left(t\right).$$ (2)

Here *L*(*t*) is an effective one-body transition amplitude and *A*_*c*_(*ω*) = −(1/*π*)Im *G*_*c*_(*t*). 3) A real-time EOM-CC approach for the cumulant core Green’s function *G*_*c*_ has been developed including excitations up to CC-singles (CCS). Intrinsic losses induced by the sudden creation of the core hole lead to shake-up effects, characterized by satellites in *A*_*c*_(*ω*), as observed in x-ray photoemission spectra (XPS). The effective one-body XAS *μ*_1_(*ω*) also builds in orthogonality corrections leading to edge enhancements, as predicted by Mahan, Nozieres and de Dominicis (Mahan, 1967). Our goal here is to review these developments and to combine the one-particle absorption spectrum with a more accurate treatment of the core Green’s function, including the complete form of the CCS cumulant, as well as the full single, doubles and triple cluster excitations in the cluster amplitude equations.

In the rest of this review, Section 2 describes the theoretical approaches used, in particular a brief introduction to the cumulant approach (Section 2.1), the real-time equation of motion, coupled cluster (RT-EOM-CC) approach (Section 2.2), the frequency space implementation of the Green’s Function coupled clusters (GFCC) approach (Section 2.3), and the application to XAS (Section 2.4). Section 3 presents results for the core binding energies of small molecules, and a comparison of the theory to XPS and XAS experimental results for NH_3_. Finally, Section 4 presents a summary and discusses future developments.

## 2 Theory

### 2.1 Cumulant Approach

Within the cumulant approximation, the core-level Green’s function is defined by an exponential expression

$$G_{c}\left(t\right)=G_{c}^{0}\left(t\right)e^{C\left(t\right)},$$ (3)

where $G_{c}^{0}\left(t\right)=-i\theta \left(t\right)e^{-i\epsilon_{c}t}$ is the independent particle Green’s function (Hartree-Fock in this paper), with single particle energy ε_*c*_, and denotes the core-level in question. *C*(*t*) is the cumulant function, which builds the correlation into the Green’s function. This cumulant can be expressed in Landau form (Landau, 1944), in terms of an excitation spectrum *β*(*ω*),

$$C\left(t\right)=\int d\omega \;\frac{\beta \left(\omega \right)}{\omega^{2}}\left[e^{-i\omega t}+i\omega t-1\right].$$ (4)

As a consequence, the cumulant Green’s function is naturally normalized, with an occupation *G*_*c*_(*t* = 0) = 1. One can also analyze *A*(*ω*) = −(1/*π*)Im *G*_*c*_(*ω*), which is the natural quantity to compare to experimental x-ray photoemission spectra, and to assess the quality of many-body correlation approximations since satellites appearing in spectral quantities such as XPS are directly related to those seen in the spectral function. The above form of the cumulant also permits a natural separation into quasiparticle and satellite contributions. Separating the terms in the expression for the cumulant above, we have

$$C\left(t\right)=-a+i\Delta t+\tilde{C}\left(t\right),$$ (5)

where *a* = *∫dωβ*(*ω*)/*ω* ^2^ is the net satellite strength, Δ = *∫dωβ*(*ω*)/*ω* is the quasiparticle shift, or core-level “relaxation energy”, and $\tilde{C}\left(t\right)$ is the remainder of the cumulant, which contains the information about the satellites. By expanding about small $\tilde{C}$, the spectral function can be obtained analytically in terms of *β*(*ω*), i.e.,

$$A_{c}\left(\omega \right)=Z_{c}\delta \left(\omega -E_{c}\right)*\left[1+A_{\text{sat}}\left(\omega \right)+\frac{1}{2}A_{\text{sat}}\left(\omega \right)*A_{\text{sat}}\left(\omega \right)+\cdots \;\right],$$ (6)

where *Z*_*c*_ = *e* ^−*a*^ is the quasiparticle renormalization, *E*_*c*_ = *ϵ*_*c*_ −Δ is the quasiparticle energy, and *A*_sat_(*ω*) = *β*(*ω*)/*ω* ^2^ is the satellite spectral function (Aryasetiawan et al., 1996).

The cumulant kernel or excitation spectrum *β*(*ω*) can be approximated in a variety of ways. The most common approximation is to expand the Green’s function to low order either in the bare Coulomb interaction, giving *β*(*ω*) in terms of the second order self-energy (Vila et al., 2020), or by expanding in terms of the screened Coulomb interaction, which produces an approximation in terms of the GW self-energy (Hedin, 1999; Guzzo et al., 2011; Zhou et al., 2015). Approximate non-linear corrections can be included using real-time TDDFT (Tzavala et al., 2020). Here, as described in the next section, we calculate the cumulant including non-linear corrections within a non-perturbative approach, by expressing the Green’s function in terms of the time-dependent EOM-CC states.

### 2.2 RT-EOM-CC Theory

Our treatment of the core-hole Green’s function *G*_*c*_(*t*) is based on the CC-ansatz for the time-evolved initial state of the system with a core-hole created at *t* = 0^+^: |Ψ_*c*_ (0)⟩ ≡|Ψ_*c*_⟩ = *c*_*c*_|Ψ⟩ where *c*_*c*_ is the core annihilation operator and |Ψ⟩ is the ground state Hartree-Fock Slater determinant:

$$|\Psi_{c}\left(t\right)\rangle \equiv N_{c}\left(t\right)e^{T\left(t\right)}|\Psi_{c}\rangle .$$ (7)

Then $G_{c}\left(t\right)=-i\left\langle \Psi_{c}|e^{i\left(H-E_{0}\right)t}|\Psi_{c}\right\rangle \theta \left(t\right)$ simply becomes

$$G_{c}\left(t\right)=-iN_{c}\left(t\right)e^{-iE_{0}t}\theta \left(t\right).$$ (8)

Calculations of *G*_*c*_(*t*) are based on the real-time equation of motion coupled cluster (RT-EOM-CC) ansatz of Schönhammer and Gunnarsson (SG) (Schönhammer and Gunnarsson, 1978), in which the time-evolution of |Ψ_*c*_(*t*)⟩ is carried out using an initial value problem and propagation via the Schrödinger equation of motion *i ∂*|Ψ_*c*_(*t*)⟩/*∂t* = *H*|Ψ_*c*_(*t*)⟩. The time-evolved wave-function |Ψ_*c*_(*t*)⟩ can then be expressed using the CC ansatz in Eq. 7. The use of a single-excitations CC ansatz is justified for a single-determinant reference state approximation due to Thouless’ theorem (Thouless, 1961). In Eq. 7 *N*_*c*_(*t*) is a normalization factor, while the CC operator *T*(*t*) is defined in terms of single, double, etc., excitation creation operators $a_{n}^{†}$, i.e.,

$$T\left(t\right)=\underset{n}{\sum }t_{n}\left(t\right)a_{n}^{†}.$$ (9)

For example, for single excitations *n* = (*i*, *a*) and $a_{n}^{†}=c_{a}^{†}c_{i}$; for double excitations *n* = *i*, *j*, *a*, *b* and $a_{n}^{†}=c_{a}^{†}c_{b}^{†}c_{j}c_{i}$; etc. As is conventional in CC, the indices *i*, *j*… refer to occupied single-particle states, *a*, *b*, … to unoccupied, and *p*, *q*, … to either occupied or unoccupied ones. Projecting the EOM with either the ground state, or with singly-excited versions of it, the equations decouple naturally, i.e.,

$$\begin{array}{c} i\;\partial \ln N_{c}\left(t\right)/\partial t=\langle \Psi_{c}|\bar{H}\left(t\right)|\Psi_{c}\rangle , \end{array}$$ (10)

$$\begin{array}{c} i\;\partial \;t_{n}\left(t\right)/\partial t=\langle n|\bar{H}\left(t\right)|\Psi_{c}\rangle , \end{array}$$ (11)

where $|n\rangle =a_{n}^{†}|\Psi_{c}\rangle$ and $\bar{H}=e^{-T}He^{T}$ is the similarity-transformed Hamiltonian. The first of these results shows that the normalization factor *N*_*c*_(*t*) is a pure exponential, so that the core Green’s function *G*_*c*_(*t*) has a natural cumulant representation $G_{c}\left(t\right)=G_{c}^{0}\left(t\right)e^{C\left(t\right)}$ with a cumulant defined as

$$C\left(t\right)=-i\int_{0}^{t}dt^{\prime }\;\left\langle \Psi_{c}|\left(\bar{H}\left(t^{\prime }\right)-E_{0}^{\prime }\right)|\Psi_{c}\right\rangle ,$$ (12)

where $E_{0}^{\prime }=E_{0}-\epsilon_{c}$, and *E*_0_ is the ground state energy. As noted above, the EOM-CC cumulant can have a Landau form (Eq. 4) (Landau, 1944) that simplifies analysis of its spectrum. The cumulant kernel or excitation spectrum *β*(*ω*) from the EOM-CC approach is given by

$$\beta \left(\omega \right)=\frac{1}{\pi }Re\int_{0}^{\infty }dt\;e^{-i\omega t}\frac{d}{dt}\left\langle \Psi_{c}|\bar{H}\left(t\right)|\Psi_{c}\right\rangle$$ (13)

**FIGURE 1:**
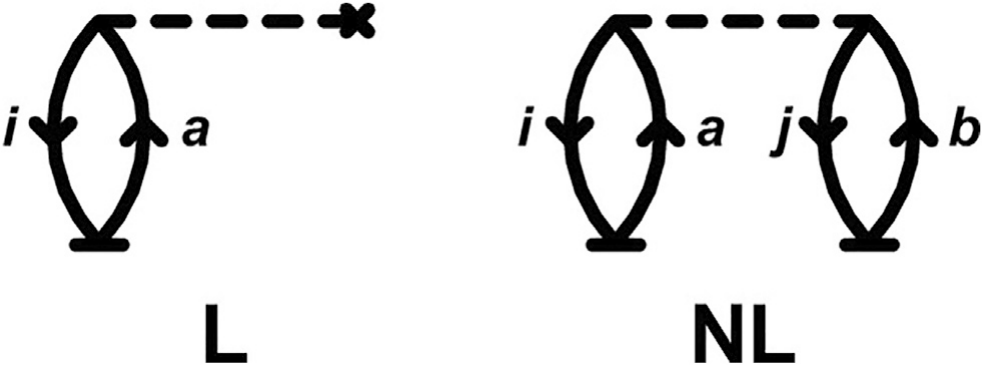
Linear (L) and non-linear (NL) CC diagrams (Crawford and Schaefer, 2000; Brandow, 1967) for the time-derivative of the cumulant in Eq. 14. Unlike in traditional EOM-CC diagrams, the base vertices (*t*) are time-dependent.

This amplitude accounts for the transfer of oscillator strength from the main peak in XPS to the satellite excitations at frequencies *ω*. The cumulant initial conditions *C* (0) = *C*′(0) = 0 guarantee the normalization of the spectral function. In addition, they ensure that its centroid remains invariant at the Koopmans’ energy −*ϵ*_*c*_. After some straightforward diagrammatic analysis to compute the matrix elements in Eqs. 10, 12, we obtain a compact expression for the time-derivative of the cumulant

$$-i\frac{dC\left(t\right)}{dt}=\underset{ia}{\sum }f_{ia}t_{i}^{a}+\frac{1}{2}\underset{ijab}{\sum }v_{ij}^{ab}t_{j}^{b}t_{i}^{a},$$ (14)

where $t_{i}^{a}=t_{n}\left(t\right)$ when *n* = (*i*, *a*), and the *f*_*ia*_ and $v_{ij}^{ab}$ coefficients correspond, respectively, to the one- and two-particle elements that define the second-quantized Hamiltonian in a core-hole reference (Vila et al., 2020). The terms on the rhs of Eq. 14 correspond to the linear- (L) and non-linear (NL) CC diagrams (Crawford and Schaefer, 2000; Brandow, 1967) shown in Figure 1. The linear term arises from the coupling of the core-hole to the *i* → *a* excitation, while the second term [which is quadratic in the amplitudes *t* (NL)] represents valence polarization effects that screen the core-hole.

Remarkably, these diagrams are completely analogous to the time-independent diagrams for the CCSD energy if only single excitations (*T*_1_) are included. It is interesting to note that only one more diagram is needed to obtain the complete CC cumulant for the core-hole Green's function, namely that from double excitations similar to the NL diagram in Figure 1, but with a cluster line joining the base vertices representing the *T*_2_ operator.

The EOM for the matrix elements of the CC amplitudes in Eq. 11 can be calculated using similar diagrammatic analysis which yield a set of coupled first-order differential equations.

$$\begin{array}{l} -i{\dot{t}}_{i}^{a}=-v_{ac}^{ic}+\left(\epsilon_{a}-\epsilon_{i}\right)t_{i}^{a} \end{array}$$ (15a)

$$\begin{array}{l} +\underset{j}{\sum }v_{jc}^{ic}t_{j}^{a}-\underset{b}{\sum }v_{ac}^{bc}t_{i}^{b}+\underset{jb}{\sum }v_{ja}^{bi}t_{j}^{b}+\underset{jb}{\sum }v_{jc}^{bc}t_{i}^{b}t_{j}^{a} \end{array}$$ (15b)

$$\begin{array}{l} +\underset{jbd}{\sum }v_{aj}^{bd}t_{i}^{b}t_{j}^{d}-\underset{jkb}{\sum }v_{jk}^{ib}t_{j}^{a}t_{k}^{b} \end{array}$$ (15c)

$$\begin{array}{l} -\underset{jkbd}{\sum }v_{jk}^{bd}t_{i}^{b}t_{j}^{a}t_{k}^{d}, \end{array}$$ (15d)

where *ϵ*_*p*_ are the bare single-particle energies. The low order terms are identical to those in our original paper (Rehr et al., 2020). However, now the complete CCS *T*_1_ approximation is used, including terms up to third order in $t_{i}^{a}$. The similarity in the form of Eq. 14 and Eq. 15 to the time-independent matrix elements used in standard CC theory implies that the overall scaling of the RT-EOM-CC approach per time step is equivalent to that of the standard CC equations of the same order per solution iteration (i.e. *N* ^4^ for CCS, *N* ^6^ for CCSD, etc.). The main difference arises in that while for the latter only a few tens of iterations are needed for convergences, RT-EOM-CC requires hundreds to thousands of steps to described the core dynamics. One final difference arises from the complex nature of the time-dependent CC amplitudes, which doubles the computational demands. Thus, approximations are highly desirable and here we review four possible levels of approximation:

1) Lowest order approximation, with only the leading terms, i.e., Eq. 15a: At this level the EOM is exactly solvable giving $t_{i}^{a}\left(t\right)=-v_{ac}^{ic}\left[\exp\left(i\omega_{ia}t\right)-1\right]$, with *ω*_*ia*_ = *ϵ*_*a*_−*ϵ*_*i*_. Moreover, the second-order self-energy cumulant (Vila et al., 2020) is obtained if only the linear part in Eq. 14 is kept. However this low level of approximation produced mean absolute errors for the core binding energies an order of magnitude larger than those with the next higher levels of approximation to Eq. 15 (2–4, as summarized below) which we have used for the results presented in Section 3.
2) Core-valence approximation: Eq. 15a together with the dominant, first four sums in Eq. 15b. This approximation includes the linear core- and valence-valence contributions and a non-linear term corresponding to excitations linked to the core-hole.
3) Full second order approximation: Keeping all terms of level 2, and the non-linear valence-valence excitations from the two sums in Eq. 15c. This gives corrections that close the gap between the QP peak and the satellites.
4) Full third order T_1_ level: This approximation retains all the terms in the T_1_ EOM in Eq. 15, including the cubic term in Eq. 15d.

Each of the approximation levels to Eq. 15 can be combined with either the linear (L), or both the linear and non-linear (NL) components of *C*(*t*) defined in Eq. 14. As we have demonstrated previously (Vila et al., 2020), the NL component is key for obtaining accurate core binding energies. Consequently it is useful to focus on the 2_*NL*_, 3_*NL*_ and 4_*NL*_ results only. For comparison results are also shown for the solution of the Dyson equation (DSE2) (Linderberg and Öhrn, 2004; Szabo and Ostlund, 1996)

$$G_{c}\left(\omega \right)={\left[1-G_{c}^{0}\left(\omega \right)\Sigma^{\left(2\right)}\left(\omega \right)\right]}^{-1}G_{c}^{0}\left(\omega \right)$$ (16)

where Σ^(2)^(*ω*) is the second order self-energy (Szabo and Ostlund, 1996); and for the frequency space Green’s function GFCCSD and GFCC-i (2, 3) methods (Peng and Kowalski, 2018a; Peng and Kowalski, 2018b).

### 2.3 GFCC Theory

In this section we briefly review the GFCC formalism introduced by Nooijen et al. (Nooijen and Snijders, 1992; Nooijen and Snijders, 1993; Nooijen and Snijders, 1995, see also Meissner and Bartlett, 1993; Kowalski et al., 2014; Bhaskaran-Nair et al., 2016; Peng and Kowalski, 2016; Peng and Kowalski, 2018a), which draws heavily on the bi-variational CC formalism (Arponen, 1983; Stanton and Bartlett, 1993) where the ground-state bra- ket (〈Ψ_*g*_|) and (|Ψ_*g*_〉) states are parametrized in a different way.

$$\begin{array}{c} \langle \Psi_{g}|=\langle \Phi |\left(1+\Lambda \right)e^{-T} \end{array}$$ (17)

$$\begin{array}{c} |\Psi_{g}\rangle =e^{T}|\Phi \rangle , \end{array}$$ (18)

where the reference function |Φ⟩ is typically chosen as a Hartree-Fock Slater determinant (for the original papers on the CC ansatz see Coester, 1958; Coester and Kümmel, 1960; Čížek, 1966; Paldus et al., 1972; Purvis and Bartlett, 1982; Paldus and Li, 1999; Bartlett and Musiał, 2007). In the above equations the *T* and Λ operators refer to the so-called cluster and de-excitation operators, respectively, which can be obtained by solving canonical CC equations for the *N*-electron system. For simplicity, in this review we will discuss the algebraic form of the retarded part of the frequency dependent CC Green’s function defined by matrix elements $G_{pq}^{R}\left(\omega \right)$:

$$G_{pq}^{R}\left(\omega \right)=\left\langle \Psi_{g}|c_{q}^{†}{\left(\omega +\left(H-E_{0}\right)-i\eta \right)}^{-1}c_{p}|\Psi_{g}\right\rangle .$$ (19)

Here *H* is the electronic Hamiltonian for the *N*-electron system, *E*_0_ the corresponding ground-state energy, *η* is a broadening factor, and *c*_*p*_ ($c_{q}^{†}$) operator is an annihilation (creation) operator for an electron in the *q*-th spin-orbital. The bi-variational CC formalism then leads to a formula for the general matrix element $G_{pq}^{R}\left(\omega \right)$ given by:

$$G_{pq}^{R}\left(\omega \right)=\left\langle \Phi |\left(1+\Lambda \right)\bar{c_{q}^{†}}{\left(\omega +{\bar{H}}_{N}-\text{i}\eta \right)}^{-1}{\bar{c}}_{p}|\Phi \right\rangle ,$$ (20)

The similarity transformed operators here $\bar{A}$ ($A=H,c_{p},c_{q}^{†}$) are defined as $\bar{A}=e^{-T}A\;e^{T}$. By defining *ω*-dependent auxiliary operators *X*_*p*_(*ω*)

$$\begin{array}{c} X_{p}\left(\omega \right)=X_{p,1}\left(\omega \right)+X_{p,2}\left(\omega \right)+\ldots \\ =\underset{i}{\sum }x^{i}{\left(\omega \right)}_{p}c_{i}+\underset{i<j,a}{\sum }x_{a}^{ij}{\left(\omega \right)}_{p}c_{a}^{†}c_{j}c_{i}+\ldots ,\;\;\forall_{p} \end{array}$$ (21)

that satisfy equations

$$\left(\omega +{\bar{H}}_{N}-\text{i}\eta \right)X_{p}\left(\omega \right)|\Phi \rangle ={\bar{c}}_{p}|\Phi \rangle ,$$ (22)

Equation 20 can then be re-expressed compactly as

$$G_{pq}^{R}\left(\omega \right)=\left\langle \Phi |\left(1+\Lambda \right)\bar{c_{q}^{†}}X_{p}\left(\omega \right)|\Phi \right\rangle .$$ (23)

The *X*_*p*_(*ω*) operators can be effectively solved using a parallel implementation of the GFCC formalism based on the approximate forms of *T*, Λ, and *X*_*p*_(*ω*) (Peng et al., 2021).

The RT-EOM-CC Green’s function differs from the frequency-space GFCC approaches (Peng and Kowalski, 2016; Peng and Kowalski, 2018b) in several respects. In particular RT-EOM-CC is based on a transformation to an initial value problem with the propagation of the *N* − 1 particle system carried out after the creation of the core-hole; in contrast the GFCC methods are implemented in frequency-space. In addition, RT-EOM-CC assumes an uncorrelated *N*-particle single-determinant ground state, while the GFCC approaches calculate this ground state using the CC ansatz (Eq. 17 and Eq. 18). Finally, the RT-EOM-CC cumulant treats the *N* − 1 particle excited states at the CCS level, while the GFCCSD approximation solves for the excitations of the *N* − 1 particle system at the approximate CCSD level, keeping only single and double excitations, as discussed near Eq. 14 of Peng and Kowalski, 2018a. Thus high order diagrams are implicitly built in the RT-EOM-CC GF from the exponential form of the cumulant (Gunnarsson et al., 1994; Lange and Berkelbach, 2018). While the RT-EOM-CC utilizes a unique approximation for the time-dependent *T*(*t*) operator, the GFCC formalisms permit the use of several levels of approximation for the *T*, Λ and *X*_*p*_(*ω*) operators (for assuring size-extensivity of diagrams defining the $G_{pq}^{R}\left(\omega \right)$ matrix elements, the “n+1” rule of Peng and Kowalski, 2018b has to be followed). The numerical complexities of the RT-EOM-CC and GFCC methods, aside from complicated tensor contractions, originate in the time propagation algorithms, and the need for solving a large number of linear equations for frequency domain, respectively. Efficient algorithms have already been tested to alleviate possible numerical problems and instabilities (Peng et al., 2019; Peng et al., 2021).

### 2.4 X-Ray Spectra

Our treatment here is adapted from that in our original EOM-CC paper (Rehr et al., 2020), but updated here with a more detailed treatment of the EOM discussed above. As outlined in the introduction, the contribution from a deep core level |*c*⟩ to the XAS is given by the time-correlation function *μ*(*t*) = *L*(*t*)*G*_*c*_(*t*) in Eq. 2, as in Nozieres and de Dominicis, 1969 and Rehr et al., 2020. The core-hole Green’s function *G*_*c*_ can be obtained from the RT-EOM-CC Eq. 8. Calculations of *L*(*t*), the one-body time-dependent transition amplitude, can be carried out using coupled equation of motion or equivalent integral equations (Langreth, 1969; Grebennikov et al., 1977; Privalov et al., 2001). Alternatively, propagation based on the overlap integrals *u*_*ij*_(*t*) can also be used, as done by Nozieres and Combescot (NC) (Nozieres and Combescot, 1971). However, it is more convenient to replace the sums over *k* with the complete set of eigenstates *κ* of the final state one-particle Hamiltonian $h^{\prime }=\sum_{\kappa }\epsilon_{\kappa }c_{\kappa }^{†}c_{\kappa }$. Then, defining the core transition operator *D* in terms of the transition matrix elements *M*_*cκ*_ = ⟨*c*|*d*|*κ*⟩, $D=\Sigma_{\kappa }M_{c\kappa }c_{\kappa }^{†}c_{c}$, the single-particle XAS amplitude *L*(*t*) becomes.

$$\begin{array}{c} L\left(t\right)=\underset{\kappa ,\kappa^{\prime }}{\sum }M_{c\kappa }^{*}M_{c\kappa^{\prime }}L_{\kappa ,\kappa^{\prime }}\left(t\right), \end{array}$$ (24)

$$\begin{array}{c} L_{\kappa ,\kappa^{\prime }}\left(t\right)=e^{i\epsilon_{\kappa }t}\left[u_{\kappa ,\kappa^{\prime }}\left(t\right)-\overset{occ}{\underset{ij}{\sum }}u_{\kappa i}\left(t\right)u_{ij}^{-1}\left(t\right)u_{j\kappa^{\prime }}\left(t\right)\right]. \end{array}$$ (25)

The leading term on the rhs of Eq. 25 can be interpreted as a contribution to *L*_*κ*,*κ*′_(*t*) from the independent particle transition amplitude for the final state when the core-hole is present *L*_0_(*t*) = Σ_*κ*_|*M*_*cκ*_|^2^  exp(*iϵ*_*κ*_ *t*), consistent with the final-state rule of von Barth and Grossman (von Barth and Grossmann, 1982). The diagonal terms *κ* = *κ*′ in Eq. 25 suppress transitions to the occupied states *κ* < *k*_*F*_ by yielding the theta function *θ*(*k*_*F*_ − *k*). The off-diagonal terms in *L*_*κ*,*κ*′_(*t*) are controlled by states with either *κ* (or *κ*′) > *k*_*F*_ or *κ*′ (or *κ*) < *k*_*F*_. Interestingly the net result can be approximated accurately by the expression

$$L\left(t\right)\approx \underset{\kappa }{\sum }|{\tilde{M}}_{c\kappa }{|}^{2}e^{i\epsilon_{\kappa }t},$$ (26)

equivalent to the one derived by Friedel (Friedel, 1969), where ${\tilde{M}}_{c\kappa }=\left\langle c|d\bar{P}|\kappa \right\rangle$, and $\bar{P}=1-\Sigma_{i=1}^{N}|i\rangle \langle i|$ projects out the occupied valence states in the ground state. Note that the sum-rule *∫dω μ*(*ω*) = *πL*(0) for the XAS is also preserved by this formula. The additional terms −Σ_*i*_⟨*c*|*d*|*i*⟩⟨*i*|*κ*⟩ from $\bar{P}$ are termed *replacement transitions* (Friedel, 1969). Physically, these terms are necessary to remove transitions into the initial occupied levels. First order perturbation theory shows that, for an attractive core-hole potential and *κ* > *i*, the integral for the overlap⟨*i*|*κ*⟩ ≈ −*v*_*ik*_/*ω*_*ik*_ < 0. Thus these terms imply an intrinsic edge enhancement factor *L*/*L*_0_ = (1 + *χ*_*κ*_) for each photoelectron level *κ* in the XAS where $\chi_{\kappa }\approx -2\Sigma_{i=1}^{N}\left(M_{ci}/M_{c\kappa }\right)\left\langle i|\kappa \right\rangle$. Though this edge-enhancement effect is non-singular in molecular systems, it is consistent with the power-law singularity $\mu_{1}∼|\left(\epsilon -\epsilon_{F}\right)/\epsilon_{F}{|}^{-2\delta_{l}/\pi }$ predicted by Mahan for metallic systems (Mahan, 1967). The XAS in Eq. 1 is finally given by a convolution of *μ*_1_(*ω*) with the spectral function *A*_*c*_(*ω*). It is convenient to shift *μ*_1_(*ω*) and *A*_*c*_(*ω*) by *ϵ*_*c*_, the energy of the core level, with *ω* = *ϵ*−*ϵ*_*c*_, so that for the non-interacting system, *μ*_1_(*ω*) reduces to the independent particle XAS. The shifted *A*_*c*_(*ω*) then accounts for the shake-up excitation spectrum

$$A_{c}\left(\omega \right)=\underset{n}{\sum }|S_{n}{|}^{2}\delta \left(\omega -\epsilon_{n}\right).$$ (27)

Here $S_{n}=\left\langle \Psi_{c}|\Psi_{n}^{\prime }\right\rangle$ is the *N* − 1, many-body overlap integral, and $\epsilon_{n}=E_{n}^{\prime }-E_{0}$ is the shake-up energy. The net effect of the spectral function *A*_*c*_(*ω*) is to broaden the XAS and significantly reduce its magnitude near the edge, transferring the weight to the satellite peaks. For metallic systems this yields an Anderson power-law singularity ${\left[\left(\epsilon -\epsilon_{F}\right)/\epsilon_{F}\left.\right)\right]}^{\alpha }$ (Nozieres and de Dominicis, 1969). This reduction effect has opposite sign to and competes with the Mahan enhancement *L*/*L*_0_ in *μ*_1_(*ω*). However, the above formulation neglects extrinsic losses and interference effects, which will likely lessen these effects. The net result, however, is a many-body amplitude correction to the independent particle XAS visible in experimental XPS. This spectrum is proportional to the spectral function *J*_*k*_(*ω*) ∼ *A*_*c*_ (*ω* − *ϵ*_*k*_), and usually measured vs photoelectron energy *ϵ*_*k*_ at fixed photon energy *ω*. Thus the peaks in the XPS correspond to a quasiparticle peak as well as satellite excitations at higher binding energies, as discussed in more detail below.

## 3 Results and Discussion

As an example of the accuracy of the RT-EOM-CCS method for core ionization energies we show results for CH_4_, NH_3_, H_2_O, HF and Ne, i.e. the ten-electron series, using the experimental geometries (NIST, 2019) for all systems (*r*_CH_ = 1.087 Å, *r*_NH_ = 1.012 Å, *a*_HNH_ = 106.67 degree, *r*_OH_ = 0.958 Å, *a*_HOH_ = 104.48 degree, *r*_FH_ = 0.917 Å), and the aug-cc-pVDZ basis set (Kendall et al., 1992). We also show spectral function and XAS results for NH_3_ for which experimental values are available in the literature. The ground state single-particle states and molecular orbitals integrals for the RT-EOM-CCS approach and the ΔSCF method were calculated from a Hartree-Fock (HF) reference, while those for the core-excited ΔSCF were derived from a spin-symmetric and occupation-constrained HF reference. The final spectra were broadened to compare with experiment as in Rehr et al., 2020 and Vila et al., 2020 with varying (1–6 eV) broadening to account for the limited basis set for the continuum; similarly XAS used constant broadening consistent with experimental broadening below and 3.5 eV above the binding energy.

**FIGURE 2:**
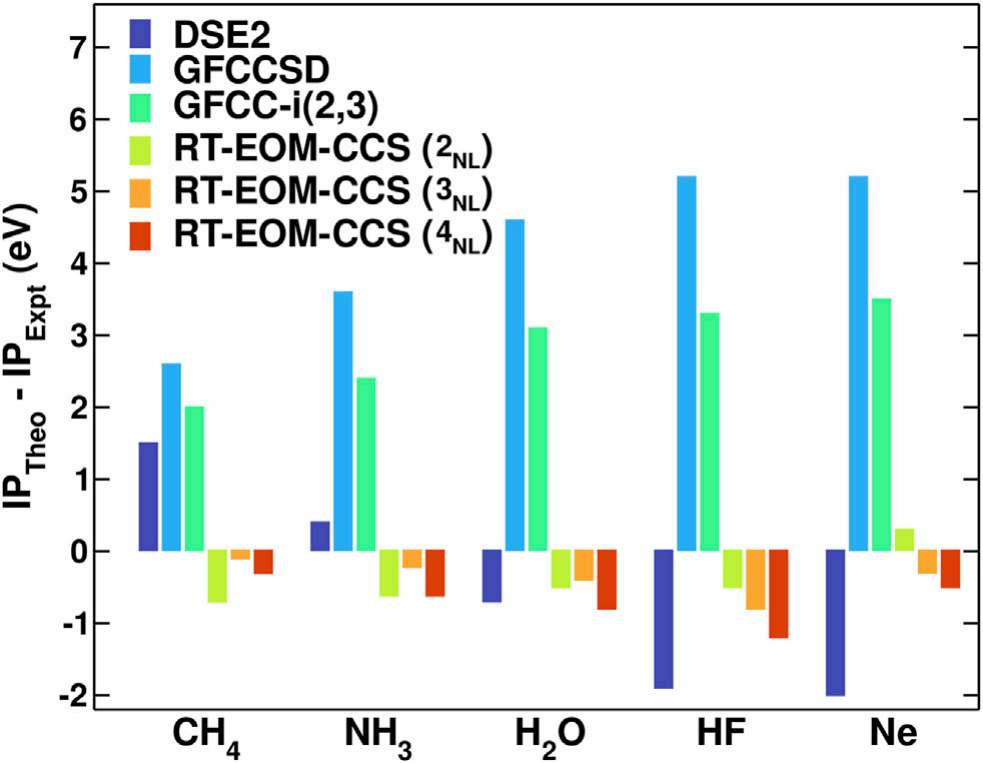
Comparison of the theory errors *vs* experiment (Karlsen et al., 2002; Buttersack et al., 2019; Viñes et al., 2018; Jolly et al., 1984; Williams, 2009) for the core binding energies. The theoretical calculations were performed with the 2-4_*NL*_ RT-EOM-CCS approximations with the aug-cc-pVDZ basis set. Also shown are results from the second order Dyson equation (DSE2) and the standard GFCCSD and GFCC-i (2, 3) coupled-cluster Green’s function methods (Peng and Kowalski, 2016; Peng and Kowalski, 2018a).

Figure 2 shows a comparison of errors vs experiment for the core binding energies of the ten-electron series molecules. The RT-EOM-CCS method shows small errors even at the simplest non-linear level of approximation (2_*NL*_), with a mean-absolute error (MAE) across systems of only 0.5 eV, significantly smaller than for the other methods tested. Although better core-optimized basis sets need to be tested to ensure full convergence with basis set size, we find (Vila et al., 2020) similar errors even for smaller basis sets (e.g. DZVP and cc-pVDZ). We emphasize that these accurate results arise from the non-linear terms in $v_{ij}^{ab}$ in the expression of the cumulant (Eq. 14), which reduce the error typically by an order of magnitude. Of the ten-electron systems, the Ne atom has the smallest MAE across the methods (0.3 eV), while for the molecules the MAE increases systematically from CH_4_ (0.4 eV) to HF (0.8 eV). It should be noted that these results do not include contributions from changes in the vibrational zero point energy (which are expected to be an order of magnitude smaller) or from relativistic effects. The latter can be significant even for these light elements (Keller et al., 2020). For instance, the inclusion of relativistic effects in the calculation of the C, N, O and F atoms increases the 1s core binding energies by 0.1, 0.3, 0.5 and 0.8 eV, respectively (Pueyo Bellafont et al., 2016). If these corrections are applied to our results the MAE are reduced by 50%.

**FIGURE 3:**
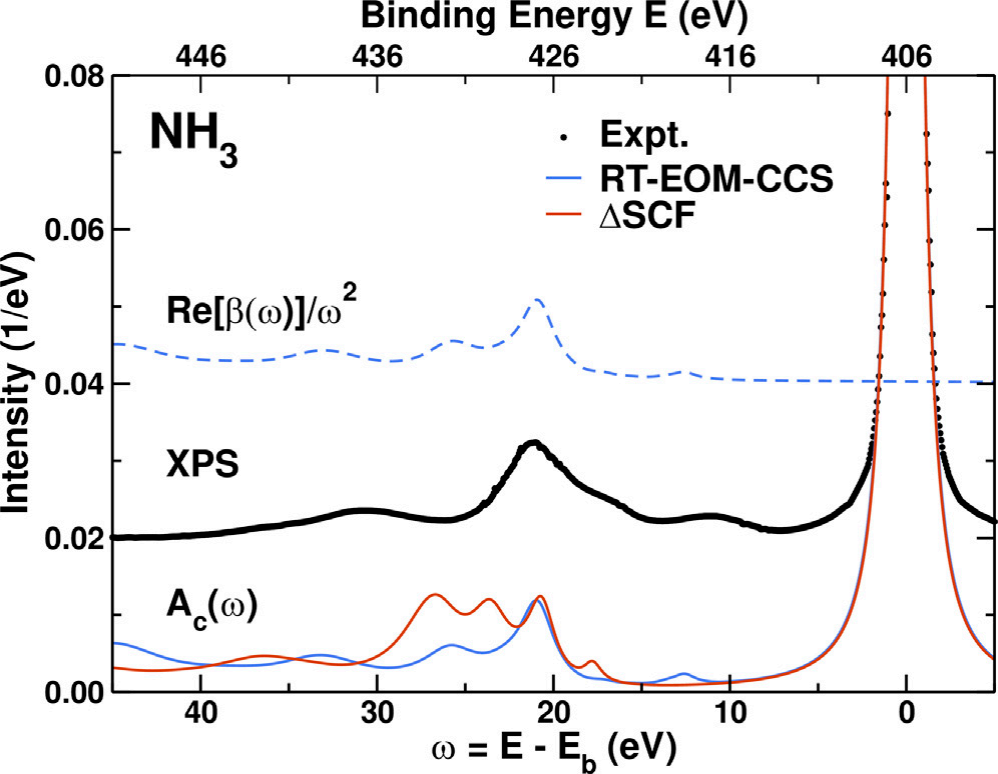
Comparison of the 4_*NL*_ RT-EOM-CCS and ΔSCF core spectral functions *A*_*c*_(*ω*) (full lines) and cumulant kernel *β*(*ω*) (dashed lines, shown only for the RT-EOM-CC method) for the NH_3_ molecule, to the experimental XPS (dots) (Sankari et al., 2006). Energies are shows in either absolute binding energy *E* or versus excitation energy *ω* = *E*−*E*_*b*_ with respect to the experimental core binding energy *E*_*b*_ = 405.52 eV (Sankari et al., 2006). All the theoretical results were obtained with the aug-cc-pVDZ basis set. The theoretical *A*_*c*_(*ω*) were broadened and include a scissors shift of 3.9 eV.

Figure 3 shows results for the spectral function *A*_*c*_(*ω*) and the cumulant kernel *β*(*ω*). These are shown vs. binding energy to compare more readily to the experimental XPS. *β*(*ω*) is dominated by shake-up excitation peaks about 20–30 eV above the quasiparticle peak that correspond precisely with the inelastic losses in *A*_*c*_(*ω*). The satellites structure is in reasonable agreement with that observed in XPS (Sankari et al., 2006) once scissors corrections are included, despite the fact that our HF-based Hamiltonian overestimates the excitation energies. From the Landau form in Eq. 4, the strength of the quasi-particle peak is defined by the renormalization constant *Z*, where for the 4_*NL*_ approximation to the EOM-CC cumulant *Z* = exp (−*a*) = 0.70. The satellite strength is *a* = *∫dω β*(*ω*)/*ω* ^2^ = 0.35. This matches the numerical integration over the QP peak that yields *Z* = 0.70, in good agreement with the ΔSCF value *Z* = 0.76. The renormalization constant *Z* is also partly responsible for the amplitude reduction factor $S_{0}^{2}$ for the XAS fine structure (Rehr et al., 1978). We also find that the RT-EOM-CCS values for *Z* agree with those obtained using the frequency-space CC Green’s function methods (Peng and Kowalski, 2016; Peng and Kowalski, 2018a). Moreover, the energy shift Δ from the middle term in Eq. 5 is the “relaxation energy,” that introduces electron-electron correlations corrections to the Koopmans’ theorem approximation of the core binding energy. Here we find that Δ = *∫dω β*(*ω*)/*ω* = 17.1 eV, with a core binding energy *E*_*b*_ = |*ϵ*_*c*_|−Δ = 406.1 eV, in good agreement with the experimental value of 405.52 eV, and the position of the quasiparticle peak in *A*_*c*_(*ω*) at 404.9 eV.

**FIGURE 4:**
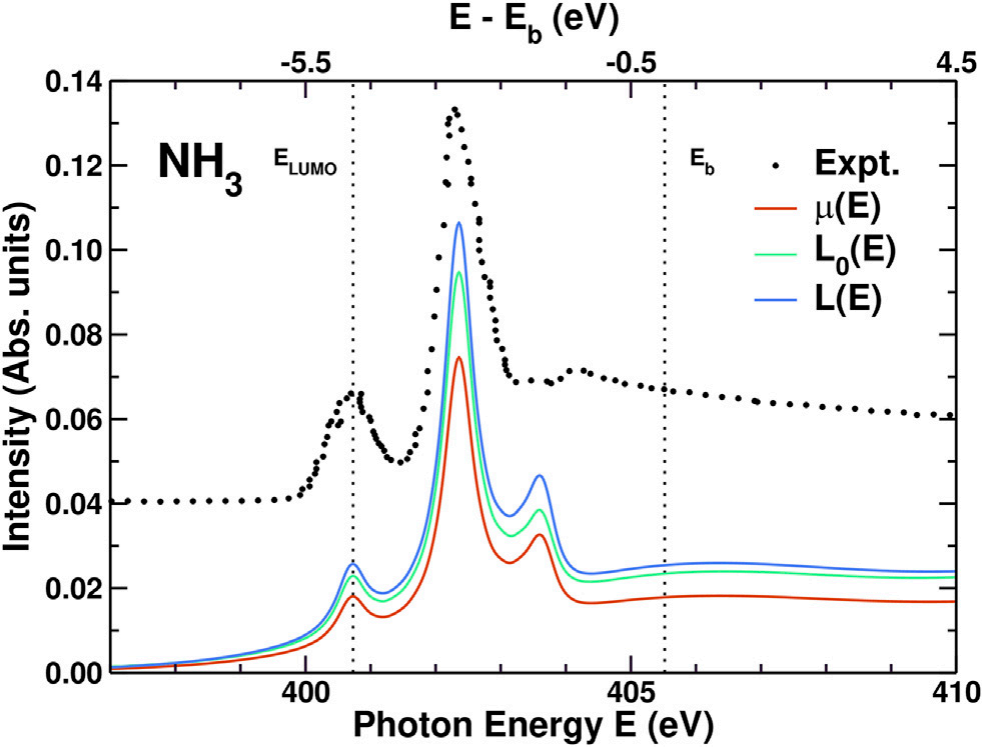
Comparison of the experimental XAS *μ*(*E*) for NH_3_ (Wight and Brion, 1974) vs photon energy *E* to those calculated from the convolution in Eq. 2, the effective one-body XAS *L*(*E*) = *μ*_1_(*E*) and the independent particle XAS *L*_0_(*E*) = *μ*_0_(*E*) from Eq. 25. The N x-ray *K* edge lies just under *E*_LUMO_ while *E*_*b*_ is the ionization threshold. In order to account for the sparsity of the Gaussian-type orbital basis set in the continuum region above *E*_*b*_, the comparison to experiment includes variable broadening (see text) that increases to a maximum of 3.5 eV above *E*_*b*_.

Results for the XAS, including experiment (Wight and Brion, 1974), are shown in Figure 4. The overall agreement between theory and experiment is quite good for the positions and relative intensities of the first two peaks. The third peak, at 403.5 eV, is almost in the continuum and is more difficult to describe with our limited basis set. For this molecule, the corrections to the independent particle XAS (*L*_0_(*ϵ*)) are clearly visible: First, the edge enhancement factor 1 + *χ* increases the intensity to *L*(*ϵ*). Second, the amplitude reduction factor from the spectral function *A*_*c*_(*ϵ*), which has opposite sign and is approximately twice as strong, reduces the intensity to the final *μ*(*ϵ*). Since the leading satellites peaks in *A*_*c*_(*ϵ*) are 20–30 eV above the QP peak, the corresponding XAS satellite features fall in the continuum and are thus not visible.

## 4 Conclusions

This review describes a combined equation of motion coupled cluster approach in real-time to calculate excitations corresponding to intrinsic losses in XAS and XPS. The approach is based on the cumulant form of the Green’s function representation for the core-hole spectral function that arises naturally from the coupled cluster ansatz. This theoretical connection between the cumulant approach, a powerful tool for computing satellites in solid state physics, to the coupled cluster approach which is the gold standard for accuracy in quantum chemistry brings together two previously mostly unrelated fields, thus opening new areas of research. Unlike our previous treatment of the XAS, where an approximate, effective single-particle Hamiltonian was used, here we use the full two-particle one, yet for simplicity we still limit the representation of the reference wavefunctions functions to single-determinants. We show that the cumulant form aids in both the physical interpretation of many-body effects observed in the spectra as well as the numerical simulations. We find that, for the XAS, a convolution form in terms of an effective single-particle spectrum and the core-hole spectral function is key to accounting for two types of many-body effects: First, inelastic losses caused by shake up excitations, accounted for the spectral function. Second the edge enhancement due to orthogonality. Both effects modulate the XAS amplitude in opposite direction near threshold, despite being non-singular for molecular systems. Interference terms and extrinsic losses from the coupling between the core-hole and the photoelectron are ignored. Nevertheless, these effects tend to cancel due to their opposite signs. The formal behavior of the RT-EOM-CC cumulant Green’s function is similar to that in other approaches, e.g., field-theoretic methods such as the linked-cluster theorem, or the quasi-boson approximation (Nozieres and de Dominicis, 1969; Langreth, 1970; Hedin, 1999). For condensed matter systems, the cumulant kernel function *β*(*ω*) is directly connected to the loss function or the screened Coulomb interaction, and represents collective excitations such as density fluctuations arising from the sudden creation of the core-hole (Langreth, 1970; Kas and Rehr, 2017). Other extensions to the approach reviewed here are feasible. For instance, an analogous treatment is possible to study x-ray emission spectra instead of XAS (Nozieres and Combescot, 1971) by changing the unoccupied single-particle states for the occupied ones. Finally, bigger systems computed with a more user-friendly and efficient implementation, including higher excitations, will be presented elsewhere.

## Data Availability

The data that support the findings of this study are available from the corresponding author upon reasonable request.
